# A deep learning model predicts the presence of diverse cancer types using circulating tumor cells

**DOI:** 10.1038/s41598-023-47805-2

**Published:** 2023-11-30

**Authors:** Somayah Albaradei, Nofe Alganmi, Abdulrahman Albaradie, Eaman Alharbi, Olaa Motwalli, Maha A. Thafar, Takashi Gojobori, Magbubah Essack, Xin Gao

**Affiliations:** 1 Computer Science Department, Faculty of Computing and Information Technology, King Abdulaziz University, 80200 Jeddah, Saudi Arabia; 2 Center of Excellence in Genomic Medicine Research, King Abdulaziz University, 21589 Jeddah, Saudi Arabia; 3 Al-Hada Armed Forces Hospital, Taif, Kingdom of Saudi Arabia; 4 College of Computing and Informatics, Saudi Electronic University (SEU), Madinah, Saudi Arabia; 5 College of Computers and Information Technology, Taif University, Taif, Saudi Arabia; 6 Computational Bioscience Research Center (CBRC), King Abdullah University of Science and Technology (KAUST), Thuwal, Saudi Arabia; 7 Computer Science Program, Computer, Electrical and Mathematical Sciences and Engineering Division (CEMSE), King Abdullah University of Science and Technology (KAUST), Thuwal, Saudi Arabia

**Keywords:** Cancer, Cancer models, Metastasis

## Abstract

Circulating tumor cells (CTCs) are cancer cells that detach from the primary tumor and intravasate into the bloodstream. Thus, non-invasive liquid biopsies are being used to analyze CTC-expressed genes to identify potential cancer biomarkers. In this regard, several studies have used gene expression changes in blood to predict the presence of CTC and, consequently, cancer. However, the CTC mRNA data has not been used to develop a generic approach that indicates the presence of multiple cancer types. In this study, we developed such a generic approach. Briefly, we designed two computational workflows, one using the raw mRNA data and deep learning (DL) and the other exploiting five hub gene ranking algorithms (Degree, Maximum Neighborhood Component, Betweenness Centrality, Closeness Centrality, and Stress Centrality) with machine learning (ML). Both workflows aim to determine the top genes that best distinguish cancer types based on the CTC mRNA data. We demonstrate that our automated, robust DL framework (DNNraw) more accurately indicates the presence of multiple cancer types using the CTC gene expression data than multiple ML approaches. The DL approach achieved average precision of 0.9652, recall of 0.9640, f1-score of 0.9638 and overall accuracy of 0.9640. Furthermore, since we designed multiple approaches, we also provide a bioinformatics analysis of the gene commonly identified as top-ranked by the different methods. To our knowledge, this is the first study wherein a generic approach has been developed to predict the presence of multiple cancer types using raw CTC mRNA data, as opposed to other models that require a feature selection step.

## Introduction

Cancer metastasis has been the primary cause of 90% of cancer deaths worldwide^1^. Metastasis is the process wherein cancer cells detach from the primary tumor and intravasate into the bloodstream to reach distant organs and develop into new tertiary or metastatic tumors. In the peripheral blood circulatory system, the cancer cells are called circulating tumor cells (CTC), and the tumor-derived fragmented DNA, circulating tumor DNA (ctDNA). Because CTCs and ctDNA are part of the cell-free circulating tumor DNA (cfDNA) acquired through non-invasive blood biopsies to detect and monitor tumors, more and more efforts are being directed toward its use to enable rapid and automatic cancer identification and classification.

Machine Learning (ML) and Deep Learning (DL) are the perfect tool sets that can harness the sheer volume of CTC, cfDNA, and ctDNA data in a high dimensional space to reveal patterns that can guide early diagnosis, understanding of metastatic spread, and drug selection^2–6^. One hallmark, population-scale studies that combine cancer cfDNA with ML is The Circulating Cell-free Genome Atlas (CCGA) study^7^. It aims to determine whether ML can detect and localize multiple cancer types with high specificity from genome-wide cfDNA sequencing data. In the first CCGA sub-study, whole-genome bisulfite sequencing (WGBS) has been found to outperform whole-genome sequencing (WGS) and targeted genome sequencing techniques concerning genome-wide methylation patterns. In the second sub-study, custom models recognize methylation patterns per region as similar to those derived from a specific cancer type. A pair of logistic regression ensembles further classify cancer/non-cancer samples and perform tissue of origin localization^8^. Building on these concepts, Li et al.^9^ have developed a novel approach named DISMIR that provides robust and sensitive cancer detection from low-depth cfDNA sequencing data. This technique integrates data from WGS and WGBS of plasma cfDNA. The novel feature engineering involved in DISMIR is the ‘switching region’ concept that effectively defines cancer-specific differentially methylated regions that aid in the source prediction of individual reads. Mapping cfDNA reads back to the source helps predict the location of cancer and tumor burden. DISMIR applies a DL model incorporating DNA sequence and methylation state to indicate the source of every read and cancer status. This model performs well for hepatocellular carcinoma detection. ML approaches and cfDNA data can assist in the early-stage detection of cancer. Wan et al.^10^ have developed computational techniques that learn associations between cfDNA profiles and cancer status and assist in classifying non-cancer controls and early-stage colorectal cancer (CRC) patients. They transformed the WGS data from cfDNA into relevant input features by counting the number of fragments overlapping each known protein-coding gene, followed by normalization to account for feature-length, read depth, and sequence-content biases.

Overall, these studies show that CTCs, ctDNA, and cfDNA data analyses is a promising field for advancing early cancer detection, management and monitoring that may prove to be of indispensable clinical use in the near future. However, many ML models designed to analyze cfDNA data are developed for specific cancer making them open to doubt for other cancer types. One possible way to overcome this is to create a model trained on pan-cancer data or develop transfer learning procedures that can effectively use and apply features from one cancer type to another. Other areas of prospective research in cfDNA analysis would be replacing the use of ML algorithms individually as models by exploring alternatives such as ensemble and hybrid models, different neural network structures (like CNN, Autoencoders, RNN), and training techniques such as Transfer learning to increase the efficiency of models in making multiple clinically relevant decisions.

Thus, in this study, we took a generic approach to predict the presence of multiple cancer types. We used CTC data from six different cancer types and ML/DL to classify the samples using a multiclass approach. Here, because the performance of ML models is significantly influenced by feature extraction and engineering techniques, we used multiple feature ranking algorithms, including Degree, Maximum Neighborhood Component (MNC), Betweenness Centrality (BC), Closeness Centrality, and Stress Centrality. However, for the DL model, we used the entire gene set. Then to mine the essential genes, we determined the features/gene set that was commonly identified by all the feature selection methods (which include the top features used in the DL model, identified by DeepLIFT) and utilized ML to show that this subset produces prediction performances similar to the complete feature set, which shows their impact in the sample distinguishing process.

## Materials and method

### Gene expression data

We downloaded CTC samples housed in ctcRbase^11^. The CTCsamples were from six cancer types, including breast cancer (BRCA), colorectal cancer (COAD), prostate cancer (PRAD), non-small cell lung cancer (LUSC), pancreatic cancer (PAAD), melanoma (SKCM) and liver cancer (LIHC) (see Table 1). We performed three preprocessing steps as described by Albaradei et al.^12^. Note that the preprocessing step involves a quality control assessment in tandem with the utilization of normalization techniques to accomplish data standardization and address batch effects. Additionally, since the number of samples is imbalanced, we used the synthetic minority oversampling technique (SMOTE) to oversample the minority class using the imbalanced-learn python library^13^. Then, the data were split five times into 70% for training and 30% for testing. We also tested our models on three external datasets from Gene Expression Omnibus (GEO), i.e., GSE153514, which includes CTC samples from castration-resistant prostate cancer patients, GSE82198, which include CTC samples from colon cancer patients and GSE144561, which include CTC samples in from pancreatic cancer patient.

**Table 1:** Statistics of the training and testing data.

Cancer type	Source	CTC sample numbers	Use
Breast cancer	ctcRbase	339	Training
Colorectal cancer	ctcRbase	18	Training
Melanoma	ctcRbase	6	Training
Non-small cell lung cancer	ctcRbase	10	Training
Pancreatic cancer	ctcRbase	19	Training
Prostate cancer	ctcRbase	89	Training
Prostate cancer	GEO (GSE153514)	9	Independent testing
Colon Cancer	GEO (GSE82198)	3	Independent testing
Pancreatic cancer	GEO (GSE144561)	17	Independent testing

### Features used by the ML and DL models

The ML prediction workflows generally include feature selection steps to avoid dealing with high dimensional data^12^. However, for DL prediction models, there is no need for explicit feature selection in their workflow. The neural network architecture learns features from the data and captures all non-linear relationships^14^.

#### Using the PPI network to identify hub genes/features for the ML models

First, we used the GeneMANIA (Gene Ontology molecular function-based weighting) Cytoscape 3.6.0 plugin^15^ to generate a physical protein–protein interaction (PPI) network. Then, we used the Cytoscape CytoHubba plugin to identify hub genes in the constructed PPI network using different local and global scoring techniques. The global technique considers the connection between the node and the entire network, while the local rank method evaluates the relationship between the node and its immediate neighbors. We used five ranking algorithms to determine the hub genes, including two local ranking algorithms, Degree, which calculates the number of adjacent nodes, and Maximum Neighborhood Component (MNC), which calculates the size of the maximum connected component. In addition, we used three global ranking algorithms Betweenness Centrality (BC), which estimates the number of the shortest paths passing through a node; Closeness Centrality, which calculates how short the shortest paths are from a node to all nodes; and Stress Centrality which calculates the absolute number of the shortest path.

Genes were ranked based on these five scoring algorithms, and the top 100 hub genes from each ranking method were shortlisted and subsequently used to develop ML models.

#### Using DeepLIFT to identify genes/features for the DL model

We used the Deep Learning Important FeaTures (DeepLIFT)^16^, which is a feature scoring algorithm to calculate the contribution scores of each neuron (genes) in the input layer of the DL model. DeepLIFT calculates a contribution score for every gene of each input sample. The obtained contribution scores express the importance of the corresponding genes for the output (prediction) layer. Then, we ranked the genes based on their importance scores and selected the top 100 ranked genes for further analyses.

### Developing ML and DL models

We created a parameter search space to evaluate different configurations for the Support Vector Machines (SVM), Random Forest (RF), k-nearest neighbor (KNN) and Deep Neural Network (DNN) models (see Table 2). We implemented the ML models, SVM, RF, and KNN, from the Scikit-learn Python library^17^. For the SVM SVC class, we employed the standard parameters, radial basis function kernel with degree = 3 and gamma = auto. We also implemented an RF model with 100 trees in the forest and a max depth of 32. We implemented the KNN model with the KNeighborsClassifier function and the number of nearest neighbors equals 5.

**Table 2:** Parameter search space for optimizing SVM, RF, KNN, and DNN models.

Algorithm	Parameter	Range
*SVM*	gamma	[‘scale’, **‘auto’**]
	kernel	[‘linear’, ‘poly’, **‘rbf’**, ‘sigmoid’, ‘precomputed’]
*RF*	n_estimators,	[1, 2, 4, 8, 16, 32, 64, **100**, 200]
	max_depth,	[1, 2, 4, 8, 16, **32**, 64, 100]
*KNN*	Number of nearest neighbors	1, 3, **5**, 7, 9, 11
*DNN*	node size in each layer	[1000, 2000, 3000, 4000, 5000, 6000, **7000**, 8000, 9000, 10000] [1000, 2000, **3000**, 4000, 5000, 6000, 7000, 8000, 9000, 10000] [100, 300, **500**, 1000, 2000]
	Activation function	[‘**relu**’, ‘tanh’, ‘sigmoid’, ‘linear’]
	Optimizers	[‘**SGD**, ‘Adam, ‘Nadam’]
	Batch size	[4, **8**, 16, 32]

Best parameters are in [bold].

For the DL model, we implemented a DNN that has three hidden layers with 7000, 3000, and 500 nodes using the Python Keras library (https://github.com/fchollet/keras). We employed the SGD algorithm with the default parameters as the optimizer and used cross-entropy to compute the loss between actual and predicted labels. We set the number of epochs to 100 and the batch size to 8. We used the early stopping and dropout (with a drop rate of 0.3) techniques to avoid overfitting.

### Bioinformatics analyses

Gene enrichment analysis was performed with Enrichr (Chen et al., 2013)^18^ via Fisher’s exact test. We used the following databases for the analysis: catalog (https://www.ebi.ac.uk/gwas/), KEGG Human database (KEGG, www.kegg.jp/kegg/kegg1.html), MGI mammalian phenotype level (https://www.informatics.jax.org/vocab/mp_ontology), and the biological process branch of gene ontology (GO:BP; http://geneontology.org/).

The analysis compared two gene lists. The first list comprises 66 genes from the union gene list generated from the five topological ranking algorithms. The second list includes the 25 genes commonly identified by the five topological ranking algorithms and DL methods. The statistically significant enriched terms were considered for the adjusted P-value < 0.01.

We also used miRNet^19^ (can be accessed from the link: https://www.mirnet.ca/miRNet/home.xhtml) to determine the critical set of microRNA associated with the 66 genes commonly identified as top-ranked by the multiple ranking algorithms used in this study. Note, we did not repeat this process for the 25 commonly identified genes, as the 25 genes are a subset of the 66 commonly identified genes.

## Results and discussion

### The study design

**Figure 1:**
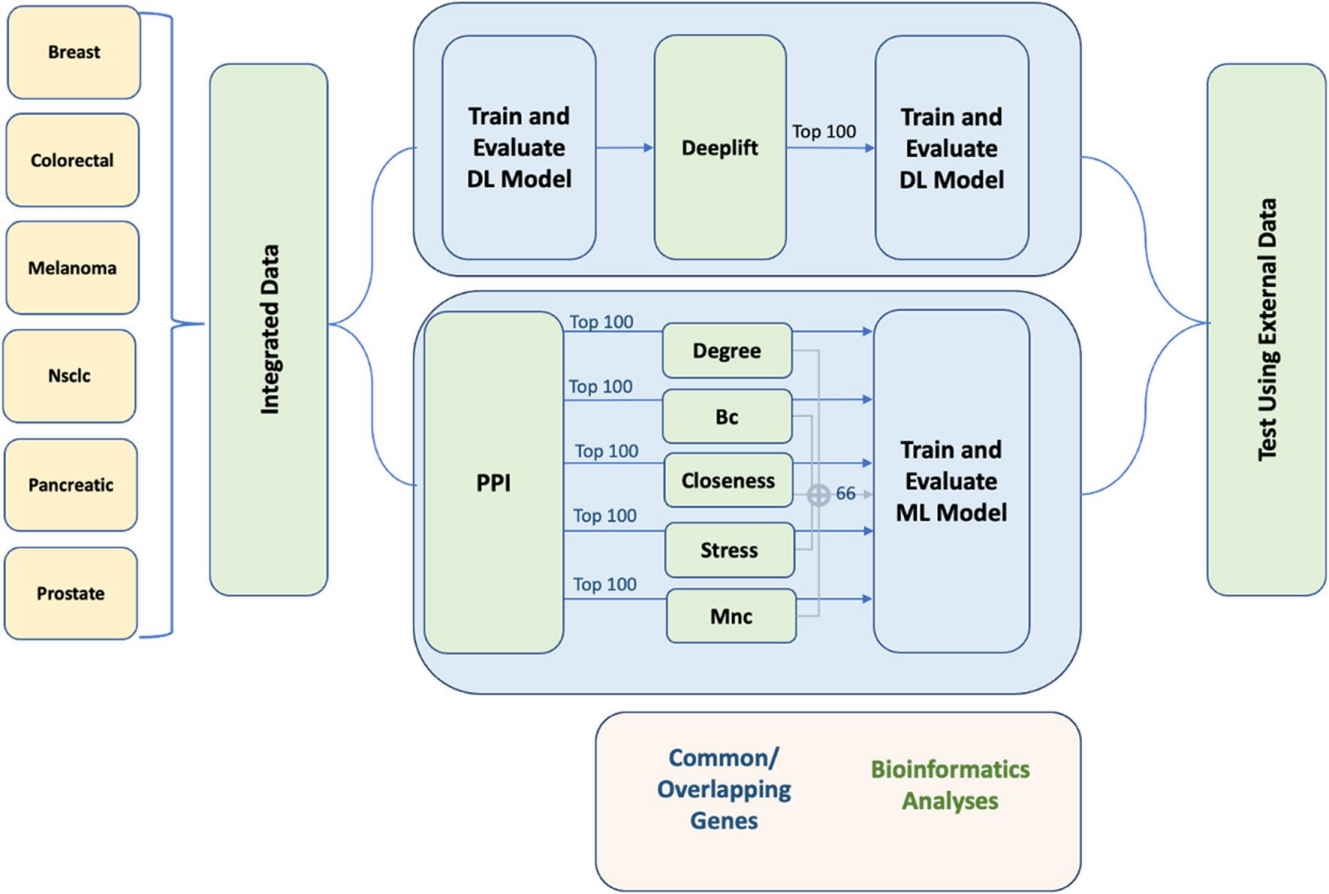
The study workflow, which consists of six main steps. Firstly, data collection. Then, the data is used to identify the top 100 hub genes/features through graph ranking algorithms for ML models, as well as for training a DL model. Next, building and evaluation of ML/DL models and test them with independent datasets. We then mine essential genes by analyzing commonly identified features/gene sets and assessing their impact using ML. Finally, we perform bioinformatics analyses on the gene set.

The workflow of our study incorporates six main steps, as depicted in Fig. 1. First, we collated 58,347 genes from 481 CTC samples retrieved from the ctcRbase^11^ database accessed in December 2022 (Table 1 provides the statistics of these datasets), which we preprocessed and applied SMOTE on to create an integrated dataset that we split into training and testing sets. Second, we used the integrated data for two objectives, (1) to identify the top 100 hub genes/features to be fed to the ML models using five graph ranking algorithms, and (2) as features (i.e., the entire gene set) to train the DL model. Third, we built and evaluated the ML/DL models using the features described in the previous step. Fourth, we tested our best models using independent datasets. Fifth, we mined the essential genes by determining the commonly identified features/gene set, then utilized ML to evaluate the impact of these genes in the sample classification process, and we performed bioinformatics analyses on the gene set.

### Evaluating the prediction performances of the ML and DL models

We evaluated the changes in the prediction performances of the ML models (SVM, RF and KNN) when fed the top 100 features (hub genes) determined by the five ranking algorithms and the DL (DNN) model when we fed the raw mRNA data directly.

Briefly, we used the 18,790 genes to construct a PPI network using GeneMANIA. As a preprocessing step, we removed all nodes (genes) with no connected edges, which resulted in a network consisting of 15,660 nodes (i.e., genes) and 159,560 edges (i.e., direct physical PPI). We fed this network into Cytoscape software to visualize and determine the hub genes using the cytoHubba plugin. Then, we obtained the 100 top-ranked hub genes for five topological ranking algorithms, including Degree, Betweenness Centrality (BC), Maximum Neighborhood Component (MNC), Closeness Centrality, and Stress Centrality (see Supplementary Table).

#### Prediction performances of models when fed hub genes determined by ranking algorithms

We developed ML models (SVM, RF, and KNN) using features (hub genes) determined by five ranking algorithms separately (see Table 3). Briefly, we first used one of the ranking algorithms to determine the top 100 ranked hub genes. Then, we trained and tested the SVM, RF, and KNN classifiers by feeding them the top 100 ranked genes. We repeated the training and testing five times using different training and testing splits and calculated various metric scores on each test set. Eventually, we aggregated the results by averaging the metric scores on the test data. We performed the same procedure for all ranking algorithms.

**Table 3:** The prediction performances of SVM, RF, and KNN when fed the top 100 hub genes determined by five ranking algorithms.

Maximum neighborhood component	Accuracy	Weighted precision	Weighted recall	Weighted F1-score
SVM	0.8993	0.904	0.8993	*0.8978*
RF	0.9424	0.943	0.9424	**0.9424**
KNN	0.7986	0.8073	0.7986	0.7912
Betweenness centrality				
SVM	0.9137	0.9299	0.9137	*0.9086*
RF	0.9353	0.9365	0.9353	**0.9349**
KNN	0.8201	0.8338	0.8201	0.8014
Degree				
SVM	0.8993	0.902	0.8993	*0.8951*
RF	0.9353	0.9393	0.9353	**0.9331**
KNN	0.777	0.7936	0.777	0.7644
Stress centrality				
SVM	0.8777	0.8849	0.8777	*0.8736*
RF	0.9281	0.941	0.9281	**0.9268**
KNN	0.7698	0.7925	0.7698	0.7541
Closeness centrality				
SVM	0.8993	0.8988	0.8993	*0.8937*
RF	0.9281	0.9432	0.9281	**0.9263**
KNN	0.7842	0.785	0.7842	0.7638

The bold and italics results indicate each ranking algorithm’s best and second-best performing models.

Table 3 provides the prediction performances of ML models fed the hub genes as features. The results show that the RF classifier achieved the best result consistently, followed by the SVM classifier, for all five sets of features determined by the ranking algorithms. The RF classifier achieved the best and second-best prediction performances with an F1-score (a combination of precision and recall metrics) of 0.9424 and 0.9349 using the MNC and BC top 100 ranked hub genes, respectively. Similarly, the SVM classifiers’ best and second-best prediction performances were also achieved with the BC (F1-score of 0.9086) and MNC (F1-score of 0.8978) top-ranked hub genes, as well as the worst-performing classifier, KNN. Thus, BC (global ranking algorithm) and MNC (local ranking algorithm) appear to be the better ranking algorithms, followed closely by Degree, while Stress and Closeness Centrality generally produced the worst performances for all the models.

#### Prediction performance of the DL model when fed the raw mRNA data directly

**Figure 2:**
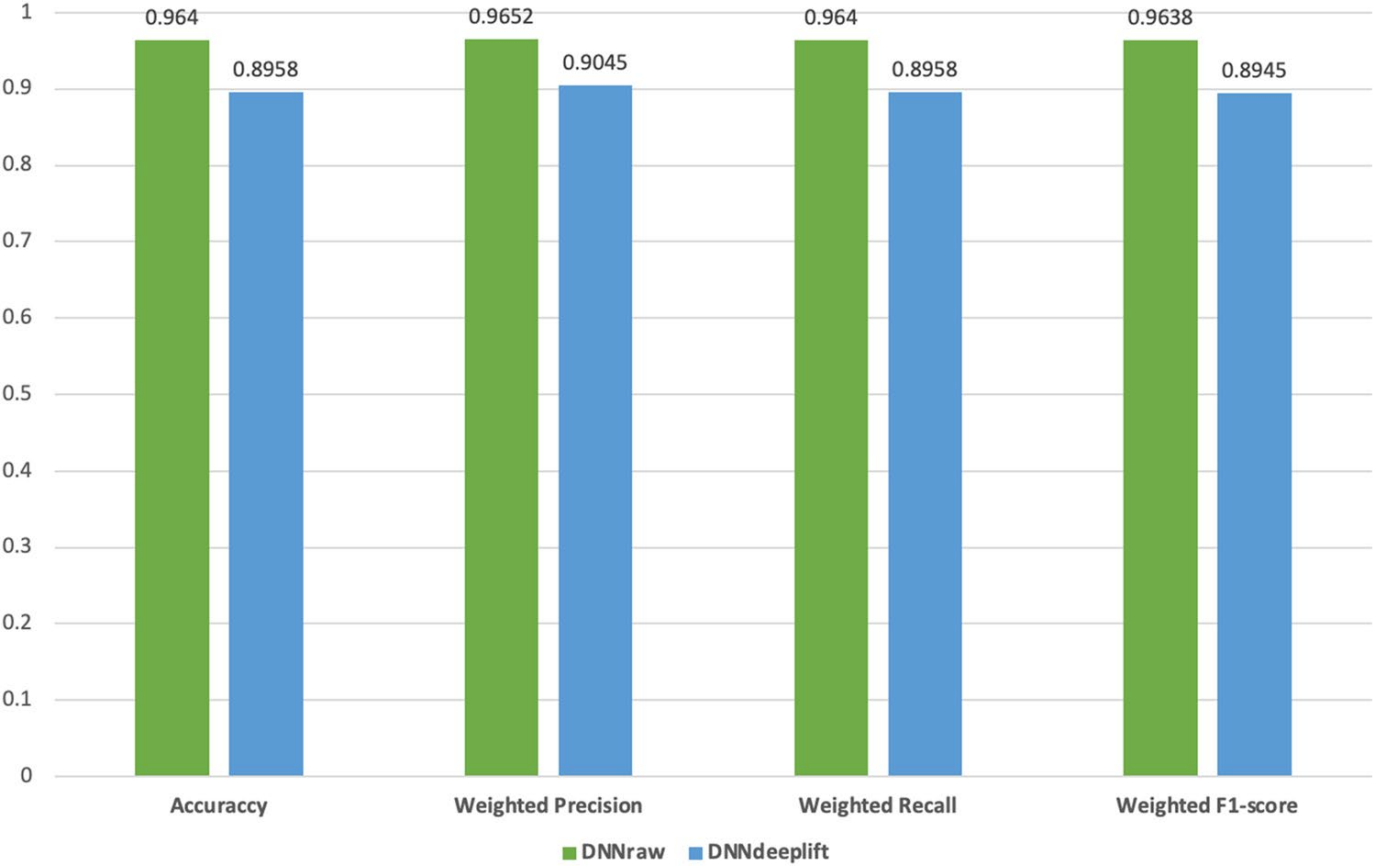
Column chart depicts the prediction performance of the DL model using (1) the entire raw mRNA data set and (2) the top 100 ranked genes determined by DeepLIFT. It is evident from the image that the DL model's prediction performance using the top 100 ranked genes is only approximately 7% lower than the performance achieved using the entire raw mRNA dataset. This striking similarity suggests that these selected genes play a crucial role in contributing to the overall performance of the DL model.

When using the DL model, DNN, we achieved average precision of 0.9652, recall of 0.9640, f1-score of 0.9638 and overall accuracy of 0.9640 (see Fig. 2). DNN performs better (around 2% higher) than the best ML model performance (RF). The result suggests that the DNN models’ way of learning allowed it to better zoom in on the mRNA features that provide the added benefit of the model displaying improved generic capabilities, i.e., to predict the origin of the tumor cell among different primary sites. Thus, we also applied DeepLIFT to calculate importance scores for each gene, which we ranked to select the top 100 ranked genes. The DNN model's prediction performance with these top 100 ranked genes was only around 7% lower than the prediction performance using the entire raw mRNA data set, suggesting that these genes are the key contributors to the DNN model’s performance. Moreover, even though we observe a slight drop in the DNN model’s performance using the top 100 ranked genes, this result is still on par with the ML models’ performances.

### Evaluating the prediction performances of the ML and DL models using independent test data

**Figure 3:**
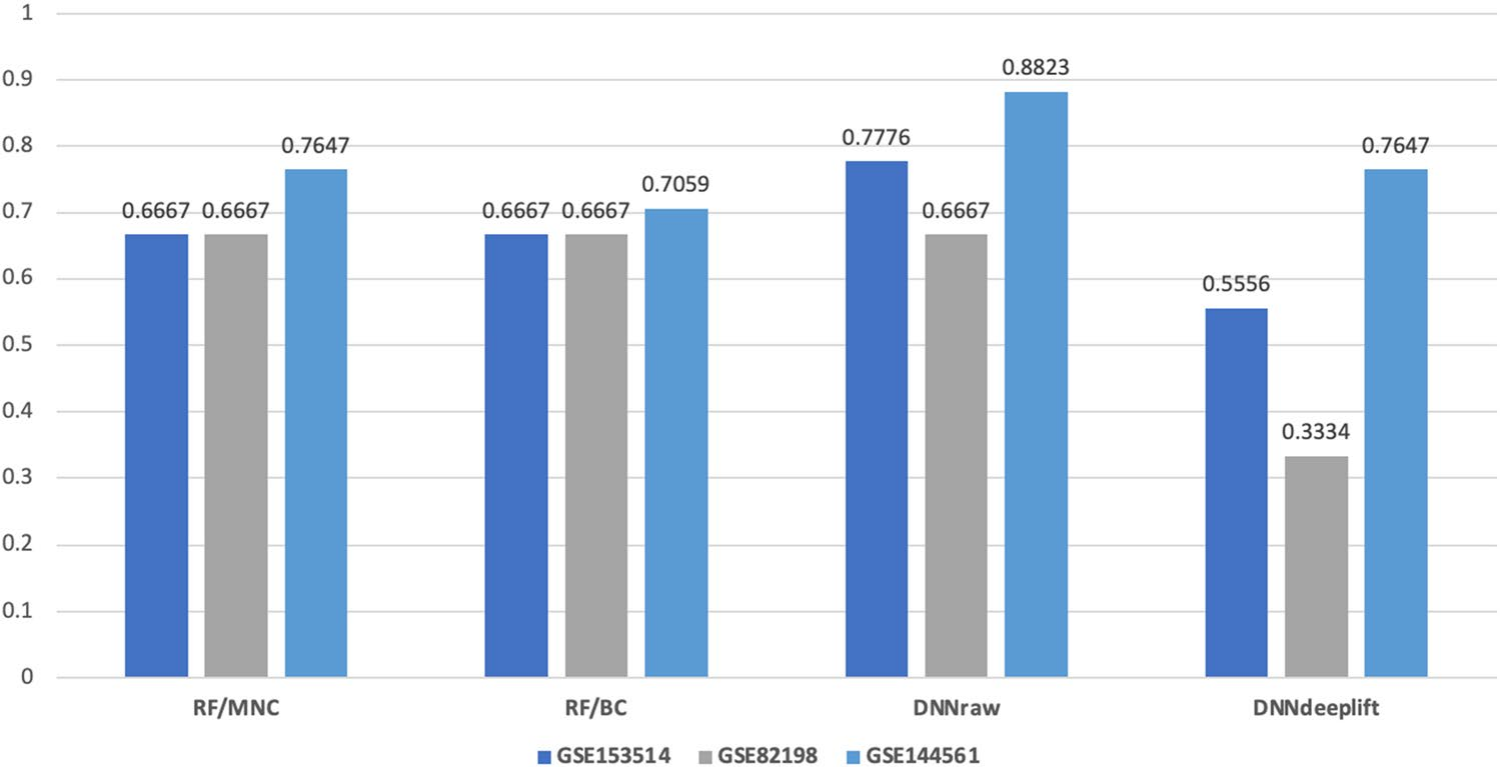
Column chart illustrating the prediction performances of the best-constructed models, RF (RF/MNC and RF/BC) and DNN (DNNraw and DNNdeeplift) on three independent datasets (GSE153514, GSE82198, and GSE144561, see Table 1). It is evident from the chart that the RF models, constructed using the top 100 ranked hub genes determined by the MNC local ranking algorithm, outperformed the RF models built with the BC global ranking algorithm. Furthermore, DNNraw achieved slightly better performances than both DNNdeeplift and the RF models across the datasets.3.4 Mining influential genes.

To further assess the robustness of our best-constructed models, RF and DNN. We tested these models on three independent datasets (GSE153514, GSE82198, and GSE144561, see Table 1). The RF models assessed include those built with the top 100 ranked hub genes determined by the best local ranking algorithm MNC, and the best global ranking algorithm BC. The RF/MNC model performed better than the RF/BC model (see Fig. 3). The RF/MNC model achieved F1-scores of 0.6667 (GSE153514, 6 out of 9 samples were classified correctly as prostate cancer and 3 were misclassified as colorectal cancer), 0.6667 (GSE82198, 2 out of 3 samples were classified correctly and 1 misclassified as breast cancer) and 0.7647 (GSE144561, 13 out of 17 samples were classified correctly as pancreatic cancer and 2 misclassified as colorectal and 2 as breast cancer) for the independent testing datasets. The RF/BC model achieved similar F1-scores of 0.6667 (GSE153514, 6 out of 9 samples were classified correctly as prostate cancer and 3 were misclassified as colorectal cancer), 0.6667 (GSE82198, 2 out of 3 samples were classified correctly and 1 misclassified as breast cancer) and 0.7059 (GSE144561, 11 out of 17 samples were classified correctly as pancreatic cancer and 3 misclassified as breast and 2 as melanoma cancer and 1 as NSCLC) but the misclassifications were different. We also assessed the DNN model built with the entire raw mRNA data set (DNNraw) and the DNN model built with the top 100 ranked genes determined by DeepLIFT (DNNdeeplift). DNNraw achieved slightly better performances than DNNdeeplift and both RF models, with F1-scores of 0.7776 (GSE153514, 7 out of 9 samples were classified correctly as prostate cancer and 2 were misclassified as colorectal and pancreatic cancer), 0.6667 (GSE82198, 2 out of 3 samples were classified correctly and 1 misclassified as breast cancer) and 0.8823 (GSE144561, 15 out of 17 samples were classified correctly as pancreatic cancer and 2 misclassified as colorectal and prostate cancer) for the independent testing datasets. DNNdeeplift achieved F1-scores of 0.5556 (GSE153514, 5 out of 9 samples were classified correctly as prostate cancer and 2 were misclassified as colorectal and 2 as pancreatic cancer), 0.333 (GSE82198, 1 out of 3 samples were classified correctly and 2 misclassified as breast cancer) and 0.7647 (GSE144561, 13 out of 17 samples were classified correctly as pancreatic cancer and 2 misclassified as breast cancer 2 misclassified as NSCLC cancer). Overall, the DNNraw model outperformed the DNNdeeplift, RF/MNC and RF/BC models, and despite the strength of the DNNraw model, the 100 top-ranked genes represented by DNNdeeplift do not achieve better prediction performance than the RF/MNC and RF/BC models.

### Identifying the influential genes using data mining techniques

The prediction performances for the RF/MNC and RF/BC models show that the best local ranking algorithm MNC, and the best global ranking algorithm BC are not zooming on the most influential genes very effectively. Thus, we here further consider if the genes commonly identified as top-ranked by all the ranking algorithms, increases the likelihood that the gene would be an influential gene.

#### Determining the influential genes based on their contribution to the prediction performances

Here, we identified the set of genes commonly identified as top-ranked hub genes by all five ranking algorithms (Degree, BC, MNC, Closeness Centrality, and Stress Centrality. Approximately two-thirds of the genes (66 genes) were commonly identified by all five ranking algorithms. Furthermore, since we also used DeepLIFT to calculate the importance scores of each gene used in the DNNraw model to identify the 100 top-ranked genes, we also determined the set of genes commonly identified by the five ranking algorithms and DeepLIFT. We found that approximately one-quarter of the genes (25 genes) were commonly identified by all five ranking algorithms and DeepLIFT.

**Figure 4:**
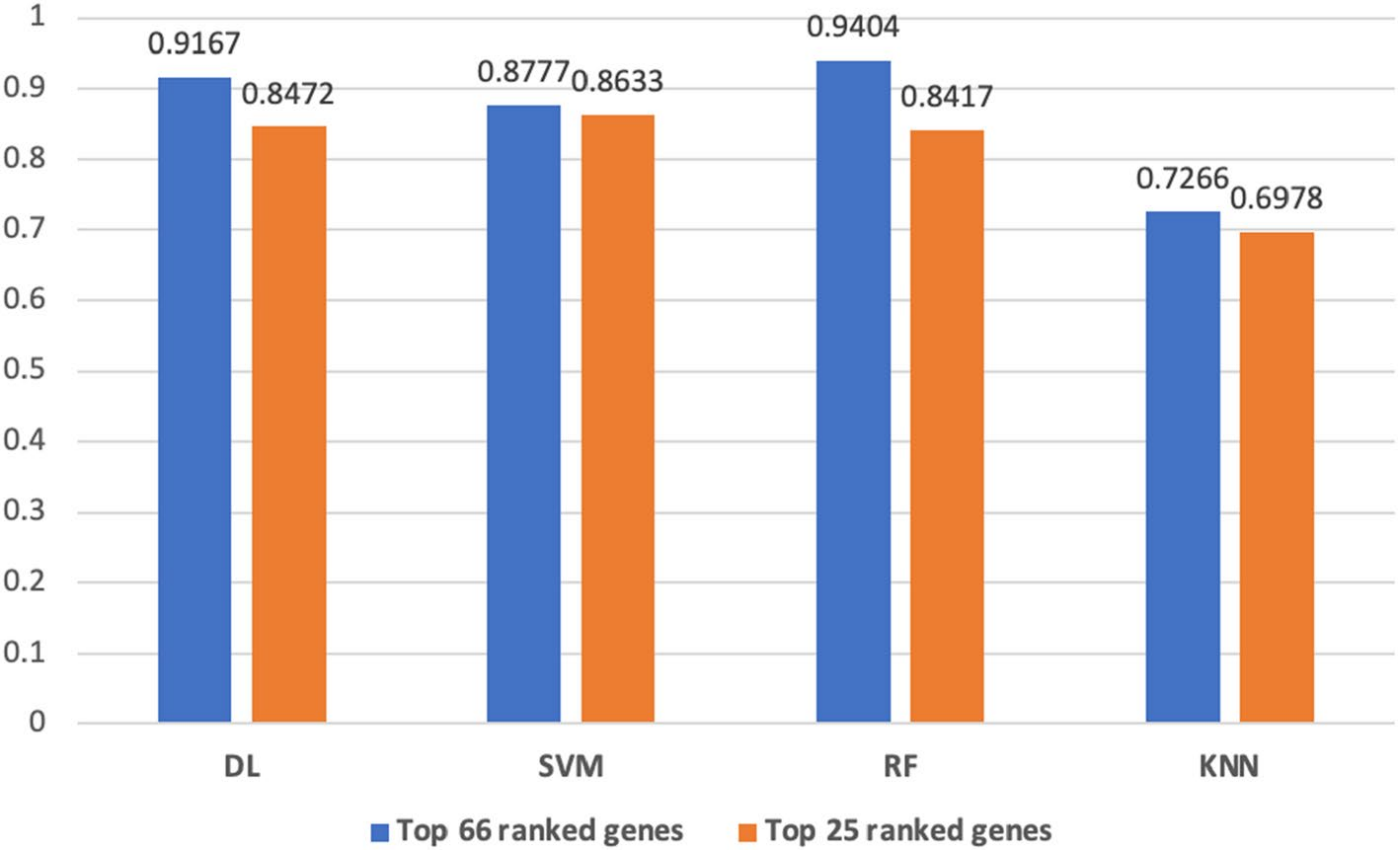
The column chart compares prediction performances among the best-performing ML and DL methods built with the 66 and 25 commonly identified top-ranked genes separately. For the models built using the 66 commonly identified genes, the RF model consistently outperforms the SVM and KNN models. Additionally, the DL model constructed with the 66 genes slightly outperforms the DNNdeeplift model. Also, Despite a decrease in performance when using the 25 top-ranked genes, the loss in performance ranges from only 0.0144 to 0.0987. This highlights the substantial impact of these 25 genes as well.

To assess if these are the influential genes, we further compare the prediction performance of the best performing DNN, SVM, RF, and KNN, with DNN, SVM, RF and KNN models built using the 66 commonly identified top-ranked genes, and the models built using the 25 commonly identified top-ranked genes (see Fig. 4). Here, for the models built using the 66 commonly identified top-ranked genes, the RF model continues to outperform the SVM and KNN models. Moreover, the RF model built using the 66 genes achieved an F1-score of 0.9404, almost identical to the RF/MNC model’s performance (F1-score of 0.9424). The DL model built with the 66 genes also slightly outperforms the DNNdeeplift model with F1-scores of 0.9167 and 0.8945, respectively. These results show that the 66 commonly identified top-ranked genes produce prediction performances identical to the performances when using the 100 top-ranked genes, which suggests the 66 genes are the influential genes. Moreover, this finding is further substantiated by the loss in performance observed for the models built using the 25 commonly identified top-ranked genes. Nonetheless, since the loss in performance of the models constructed using the 25 genes only ranges between 0.0144 and 0.0987, this, too, shows the substantial impact of the 25 genes.

#### Bioinformatics analyses of the commonly identified top-ranked genes

We further conducted an enrichment study focused on the commonly identified top-ranked genes. Table 4 lists the top 10 GO phrases associated with the 66 hub genes commonly identified by the five ranking algorithms as top-ranked. The GO terms were related to body size, embryonic lethality, abnormal cell cycle, decreased fibroblast proliferation, and decreased immature B cell number for the MGI Mammalian phenotype database; regulation of the apoptotic process, DNA damage response, and protein modification for GO biological process database; and cancer pathway, thyroid hormone signaling pathway, PI3K-Akt signaling pathway, and Estrogen signaling pathway for the KEGG database. No significant GO terms were detected using the GWAS catalog database. Of the 66 hub genes, 24 genes function in the ‘regulation of apoptotic process’; 22 genes in ‘decreased body size’ and ‘negative regulation of the apoptotic process’; and 20 genes in ‘pathways in cancer’. The top significant terms across the four databases used in this analysis relate to 'embryonic lethality (MP:0011096)’ with an adjusted P-value of 1.7e−16. Considering the KEGG databases, the top significant GO term is ‘cancer pathways’ with an adjusted P-value of 4e−17. Additionally, 5 of the top 10 significant terms for the KEGG databases are cancer pathway related, including ‘endometrial cancer’, ‘breast cancer’, ‘prostate cancer’, ‘proteoglycans in cancer’, and ‘pathways in cancer’. Table 5 provides the top 20 genes involved in cancer pathways based on enrichment analysis using the KEGG database. In the Supplementary Material, we provide complete information on the enrichment analysis results, including the bar plots for enrichment analysis and the top 20 significant GO terms detected from each database.

**Table 4:** Enrichment analyses showing the top 10 significant GO terms associated with the 66 hub genes commonly identified as top-ranked by five ranking algorithms.

Term	Overlap	P.value	Adjusted P.value	Database
MP:0011096 embryonic lethality between implantation and somite formation, complete penetrance	10/282	2,79E−08	5,26E−06	MGI Mammalian_Phenotype
MP:0011098 embryonic lethality during organogenesis. complete penetrance	14/656	2.33E−08	5.26E−06	MGI Mammalian_Phenotype
MP:0002169 no abnormal phenotype detected	24/1944	4.82E−09	1.75E−06	MGI Mammalian_Phenotype
MP:0001698 decreased embryo size	14/537	1.84E−09	8.91E−07	MGI Mammalian_Phenotype
MP:0003077 abnormal cell cycle	10/115	4.27E−12	3.10E−09	MGI Mammalian_Phenotype
MP:0001265 decreased body size	22/1111	3.43E−12	3.10E−09	MGI Mammalian_Phenotype
MP:0003984 embryonic growth retardation	12/595	4.89E−07	5.47E−05	MGI Mammalian_Phenotype
MP:0011100 preweaning lethality, complete penetrance	18/1400	3.95E−07	4.78E−05	MGI Mammalian_Phenotype
MP:0002083 premature death	16/997	1.05E−07	1,39E−05	MGI Mammalian_Phenotype
MP:0011092 embryonic lethality, complete penetrance	11/381	4,36E−08	6,34E−06	MGI Mammalian_Phenotype
MP:0008215 decreased immature B cell number	6/57	3,25E−08	5,26E−06	MGI Mammalian_Phenotype
MP:0,011,704 decreased fibroblast proliferation	7/96	2.91E−08	5.26E−06	MGI Mammalian_Phenotype
MP:0001262 decreased body weight	20/1471	2.89E−08	5.26E−06	MGI Mammalian_Phenotype
Pathways in cancer	20/530	2.68E−16	4.83E−14	KEGG_human database
Cell cycle	11/124	2.67E−13	2.40E−11	KEGG_human database
Prostate cancer	10/97	7.54E−13	4.53E−11	KEGG_human database
Proteoglycans in cancer	12/201	2.34E−12	1.05E−10	KEGG_human database
Thyroid hormone signaling pathway	10/116	4.66E−12	1.68E−10	KEGG_human database
PI3K-Akt signaling pathway	14/354	7.73E−12	2.32E−10	KEGG_human database
Estrogen signaling pathway	10/137	2.48E−11	6.39E−10	KEGG_human database
Breast cancer	10/147	5.01E−11	1.13E−09	KEGG_human database
Hepatitis C	10/155	8.48E−11	1.70E−09	KEGG_human database
Endometrial cancer	7/58	8.09E−10	1.46E−08	KEGG_human database
signal transduction involved in mitotic G1 DNA damage checkpoint (GO:0072431)	9/63	5.41E−13	7.66E−11	GO Biological Process
ERBB2 signaling pathway (GO:0038128)	8/39	5.14E−13	7.66E−11	GO Biological Process
DNA damage response, signal transduction by p53 class mediator resulting in cell cycle arrest (GO:0006977)	9/62	4.65E−13	7.66E−11	GO Biological Process
positive regulation of cell cycle arrest (GO:0071158)	10/82	1.33E−13	2.70E−11	GO Biological Process
DNA damage response, signal transduction by p53 class mediator (GO:0030330)	10/82	1.33E−13	2.70E−11	GO Biological Process
regulation of apoptotic process (GO:0042981)	24/815	3.88E−17	1.10E−14	GO Biological Process
negative regulation of programmed cell death (GO:0043069)	19/408	3.62E−17	1.10E−14	GO Biological Process
protein modification by small protein removal (GO:0070646)	17/261	7.77E−18	3.67E−15	GO Biological Process
protein deubiquitination (GO:0016579)	17/257	5.98E−18	3.67E−15	GO Biological Process
negative regulation of apoptotic process (GO:0043066)	22/485	1.23E−19	1.74E−16	GO Biological Process

**Table 5:** The top 20 genes from among the 66 hub genes involved in cancer pathways based on enrichment analysis using the KEGG database.

Gene	Position	Pathway in cancer	References
*NTRK1*	1:156,815,640–156,881,850	Have been observed in several epithelial cancers	^20^
*GSK3B*	3:119,821,321–120,094,994	Inhibition of *GSK3B* caused tumor shrinkage in mice	^21^
*HSP90AA1*	14:102,080,742–102,139,699	Could serve as a biomarker for cancer	^22^
*EGLN3*	14:33,924,227–34,462,774	Associated with the growth of various cancers	^23^
*HSP90AB1*	6:44,246,166–44,253,888	*HSP90AB1* methylation appears to regulate the proliferation of cancer cells	^24^
*CUL1*	7:148,697,914–148,801,110	Promotes breast cancer metastasis	^25^
*FN1*	2:215,360,440–215,436,073	Associated with immune Infiltrates in Thyroid Cancer	^26^
*PIK3R1*	5:68,215,740–68,301,821	Associated with breast cancer	^27^
*ESR1*	6:151,656,691–152,129,619	Associated with breast cancer	^28^
*EGFR*	7:55,019,017–55,211,628	A driver of tumorigenesis	^29^
*ESR2*	14: 64,084,232–64,338,112	Associated with breast cancer	^30^
*MYC*	8: 127,735,434–127,742,951	Hallmark molecular feature of both the initiation and maintenance of tumorigenesis	^31^
*TRAF6*	11: 36,483,769–36,510,272	Associated with colon cancer	^32^
*MDM2*	12: 68,808,177–68,845,544	The gene amplification is associated with human tumors	^33^
*EP300*	22: 41,092,592–41,180,077	Two missense sequence alterations in *EP300* were identified in epithelial malignancies	^34^
*CTNNB1*	3: 41,194,741–41,260,096	His mutations occur in a wide spectrum of cancers	^35^
*GRB2*	17: 75,318,076–75,405,709	Overexpressed in breast cancer patients	^36^
*CALM1*	14: 90,396,502–90,408,268	Overexpressed in a wide spectrum of cancers	^37^
*TP53*	17: 7,661,779–7,687,538	His somatic mutation is the most frequent alteration in human cancers	^38^
*BIRC*	2: 32,557,703–32,557,847	Associated with breast cancer	^39^

We also conducted GO enrichment for the 25 genes commonly identified by the five ranking algorithms and DeepLIFT. Table 6 lists the top 10 GO phrases associated with the 25 genes. The enriched GO phrases include GO phrases related to cancer and pathways such as ‘Bladder cancer’, ‘Breast cancer’, ‘Transcriptional misregulation in cancer’, ‘MicroRNAs in cancer’, and ‘PI3K-Akt signaling pathway’ similar to the 66 genes. However, for the 25 genes, GO phrases related to infection such as ‘Epstein-Barr virus infection’ and ‘Kaposi sarcoma-associated herpesvirus infection’ are also enriched. This is interesting, as studies have shown that infections can lead to uncontrolled metastasis in mammalian cells by activating various signaling cascades^40–42^. For example, Lee et al.^40^ demonstrated the downregulation of the epithelial tight junction protein E-cadherin in gastric cancer cells infected with *H. pylori* cytotoxin-associated gene A (CagA). GSK-3 which induces the degradation of oncogenic proteins such as Snail, c-Myc, and Mcl-1 is also reduced with CagA infection. These results showed that CagA infection facilitates the transcriptional repressor, Snail, to suppress E-cadherin, which leads to EMT and metastasis. They also used the chorioallantoic membrane (CAM) assay to show CagA induces non-invasive MCF-7 cells to exhibit in-vivo invasive progression^40^. Chow et al.^41^ showed non-small lung cancer cells infected with *E. coli* also exhibit increased cell adhesion, migration and metastasis via TLR4 signaling. Moreover, Wynendaele et al.^42^ demonstrated that bacterial quorum sensing peptides activate the Ras/Raf/MEK/MAPK, PI3K/Akt, and STAT intracellular signaling cascades in mammalian cells. They further show bacterial quorum sensing peptide upregulates HIST1H4, and observed EGFR hyperphosphorylation and activation of Smad2/Smad3 protein linked with cell cytoskeleton rearrangement and cell migration. These results confirm that infection leads to genetic alterations and cancer metastasis through several signaling cascades, which includes 'PI3K-Akt signaling pathway', another GO phrase enriched for the 25 genes.

**Table 6:** Enrichment analyses show the top 10 significant GO terms associated with the 25 genes commonly identified by the five ranking algorithms and DeepLIFT as top-ranked.

Term	Overlap	P.value	Adjusted.P.value
Epstein-Barr virus infection	3/201	0.00173217829406245	0.00936950986333778
Transcriptional misregulation in cancer	3/186	0.00138718534528688	0.00786071695662564
Kaposi sarcoma-associated herpesvirus infection	3/186	0.00138718534528688	0.00786071695662564
Bladder cancer	2/41	0.00109974258926059	0.00688786148010578
Cellular senescence	3/160	0.000898837053270531	0.0061596964658685
Necroptosis	3/162	0.00093171879315658	0.0061596964658685
PI3K-Akt signaling pathway	4/354	0.000774761907606608	0.00576229168782415
Breast cancer	3/147	0.000703045712688142	0.00557749598732593
Ubiquitin mediated proteolysis	3/137	0.000572734632874556	0.00486824437943372
MicroRNAs in cancer	4/299	0.000410805397815705	0.00376044941077453

To further determine the key microRNA associated with the 66 and 25 commonly identified genes, we used miRNet^19^. For the 66 genes, we used a betweenness filter of 14,800 to obtain the top 10 miRNA. Subsequently, we used the 'Function Explorer' in miRNet to obtain the diseases, functions, and clusters significantly associated with the identified miRNA.

**Figure 5:**
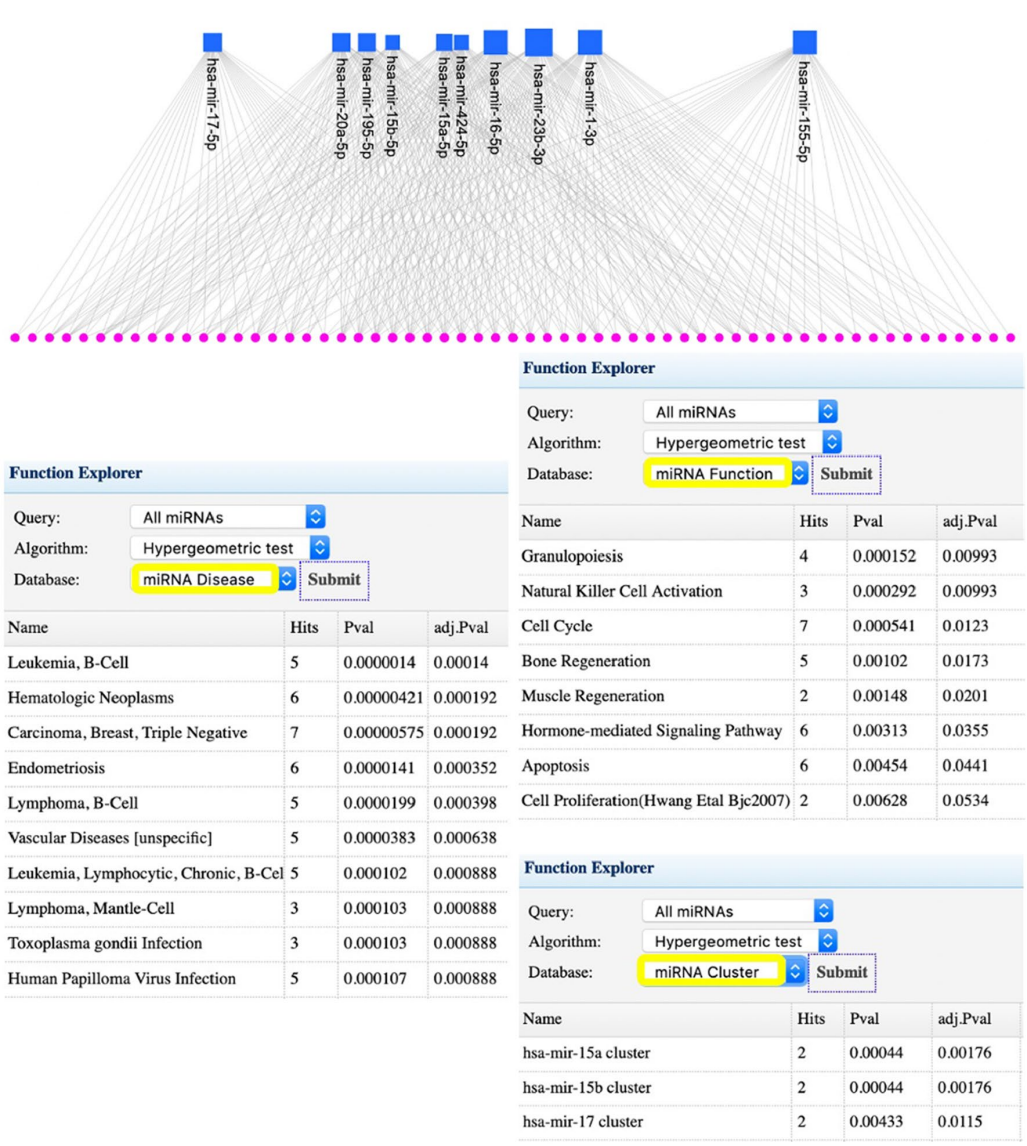
Network generated by miRNet. It shows 10 important miRNAs, represented by blue squares, that are predicted to target the 66 hub genes (represented by pink circles) commonly identified by the five ranking algorithms as top-ranked.

Figure 5 provides the network generated with miRNet that shows ten important miRNA (hsa-mir-155-5p, hsa-mir-1-3p, hsa-mir-23b-3p, hsa-mir-16-5p, hsa-mir-424-5p, hsa-mir-15a-5p, hsa-mir-15b-5p, hsa-mir-195-5p, hsa-mir-20a-5p, hsa-mir-17-5p) predicted to interact with the CTC genes.

The diseases significantly associated with the ten miRNA include Leukaemia, a cancer of the blood-forming tissue that includes the bone marrow and lymphatic system, and Hematologic neoplasms, which are neoplasms arising from hematopoietic cells found in the bone marrow, spleen, lymph nodes and peripheral blood, which ties in with the enriched functions, ‘Granulopoiesis’, and ‘Natural Killer Cell Activation’. Infectious diseases, such as ‘Toxoplasma gondii infection’ and ‘Human Papilloma Virus Infection’, were also enriched.

Interestingly, three miRNA clusters also surface in this analysis, including the hsa-mir-15a (15a/16-1) cluster, the hsa-mir-15b cluster (15b/16-2), and the hsa-mir-17 cluster. In this regard, the 15a/16 cluster in particular, is known to function as tumour inhibitors, and a substantial amount of research implicates the 15a/16-1 cluster in tumor invasion and metastasis^43–46^. Moreover, there is growing evidence that the miR-15a/16-1 cluster affects drug sensitivity and resistance^47–49^, specifically, lower miR-15a/16-1 expression increases drug resistance, while its overexpression enhances sensitivity to anticancer drugs. Moreover, the miR-15a/16 cluster has also shown promise for diagnosis and prognosis. Zidan and colleagues^50^ showed prostate cancer patients had decreased levels of serum miR-15a/16-1 compared with controls [healthy, benign prostate hyperplasia and chronic prostatitis patients] and low miR-15a/16-1 is related to higher Gleason score, tumor stage and greater lymph node involvement and metastasis. Much less research focuses on the 15b/16-2 cluster, but we do know it functions similar to the miR-15a/16-1 cluster as tumour inhibitors^51^.

On the other hand, the hsa-mir-17 cluster (also called the miR-17-92a-1 cluster) includes seven miRNAs, of which hsa-mir-17-5p and hsa-mir-20a-5p were predicted to be miRNAs interacting with genes expressed in CTC. Contrary to hsa-mir-15a-5p and hsa-mir-15b-5p, hsa-mir-17-5p is shown to elevated in different cancer types and metastasis^52–54^. Moreover, Stoen and colleagues^55^ showed high expression of miR-17-5p in tumor epithelium to be a good predictor for poor prognosis in prostate cancer patients.

## Concluding remarks

The detection and analysis of CTCs offer invaluable real-time insights into tumor evolution. They serve as a blood-based biomarker for early tumor diagnosis, disease recurrence, and metastatic spread and also a possible avenue for gauging therapeutic response and developing personalized medicine. However, there are several challenges in CTC data analysis. CTCs are rare, with a frequency of one CTC per billion normal blood cells^56^. They also have a short half-life^57^. CTCs originating from different cancer types vary significantly in size, seeding potential, and cell surface marker expression^58^. Enumerating CTCs is an arduous task prone to user bias, but it holds prognostic value, and the additional characterization of these cells can corroborate clinically relevant and treatment-specific acumen. On another hand, ML techniques, compared to traditional statistical analysis, offer objectivity, rapid execution, the ability to overcome noise, flexibility, and reduced human intervention in analyzing CTCs data. Using DL on gene expression can provide insights into tumor biology and improve our understanding of cancer biology. It can help identify key genes and pathways that are altered in different cancer types, which could reveal new targets for drug development.

This study used CTC samples from six cancer types: breast, colorectal, prostate, non-small cell lung, pancreatic, melanoma, and liver cancer to build ML and DL models that we tested on three external Gene Expression Omnibus (GEO) datasets. Feature selection was used in ML and DL prediction workflows. In ML, the PPI Network was used to generate a physical protein–protein interaction (PPI) network, and the top 100 hub genes were ranked using the five ranking algorithms. While DeepLIFT was used to identify genes for the DL model, calculating contribution scores for each neuron in the input layer. The top hub genes chosen by the five ranking algorithms were used in the study to create ML models (SVM, RF, and KNN). The SVM classifier came in second place, with the RF classifier consistently producing the best results. The MNC and BC top 100 ranked hub genes provided the best and second-best prediction results, respectively.

On the other hand, the Deep Neural Network model achieved an average precision of 0.9652, recall of 0.9640, f1-score of 0.9638, and overall accuracy of 0.9640. Therefore, it offered significantly improved generic capabilities and performed better than the best-performing ML model. We further assessed the robustness of two best-constructed RF and DNN models using three independent datasets. RF/MNC and RF/BC models achieved acceptable prediction performances, with F1-scores of 0.6667 and 0.7647, respectively. The DNN models, constructed from the whole raw mRNA data set (DNNraw) and the top 100 genes as determined by DeepLIFT (DNNdeeplift), achieved acceptable prediction performances. However, the DNNraw model performed better than the DNNdeeplift, RF/MNC, and RF/BC models. It is important to note that despite the strength of the DNNraw model, the 100 top-ranked genes represented by DNNdeeplift did not achieve better prediction performance than the RF/MNC and RF/BC models.

Enrichment analysis was performed on the hub genes, which showed that they were significantly involved in body size, embryonic lethality, abnormal cell cycle, decreased fibroblast proliferation, decreased immature B cell number, cancer-related pathways like bladder cancer, breast cancer, transcriptional misregulation, microRNAs, and the PI3K-Akt signaling pathway as revealed by GO analysis. The enrichment of the PI3K–AKT signaling pathway is commonly observed in many human cancers, including breast, lung, ovarian, and prostate. However, this pathway activation time varies among cancer types and patients. These findings underscore the crucial role of PI3K-Akt-related genes in classifying the metastasis tumor cells^59^. Moreover, GO phrases related to infection, such as Epstein-Barr virus infection and Kappi sarcoma-associated herpesvirus infection, were also enriched. Studies have shown that infections can lead to uncontrolled metastasis in mammalian cells through activating various signaling cascades. For example, CagA infection downregulates E-cadherin, GSK-3, and oncogenic proteins, leading to EMT and metastasis. Bacterial quorum sensing peptides activate intracellular signaling cascades, upregulating HIST1H4, and EGFR hyperphosphorylation. These findings confirm that infection leads to genetic alterations and cancer metastasis through various signaling cascades, and this finding being picked up by our models suggests that preventing infection in cancer patients may be key to preventing cancer progression to metastasis.

Despite the potential advantages of using DL on gene expression using cfDNA, this approach has several limitations. One major challenge is the lack of standardization in collecting, processing, and analyzing cfDNA samples, leading to significant variability between different studies. Therefore, establishing standards and protocols for sample collection, processing, and analysis is necessary. Another area for improvement is that more sensitive and precise analytical techniques are required to ensure the most minuscule amounts of cfDNA in the blood are detectable. Another challenge is the dependence of DL models on existing data for training, and there needs to be more diverse and representative datasets for cfDNA analysis. Datasets should be large and diverse enough to include multiple cancer types, cancer subtypes, and different stages of cancer for the development of robust DL models.

Our model overcomes one of these limitations through the use of raw unprocessed data, and in future work, we intend to integrate multi-omics datasets such as proteomic, epigenetic, and transcriptomic data with DL models to enable innovative biomarker discovery.

### Supplementary Information

Supplementary Figure 1.

Supplementary Table 1.

## Data Availability

In this study, we used publicly available gene expression datasets. These datasets can be found on Gene Expression Omnibus, https://www.ncbi.nlm.nih.gov/geo/ and in ctcRbase. Doi: 10.1093/database/baaa020.
