# Supplementary material for: A deep learning model predicts the presence of diverse cancer types using circulating tumor cells
Source: Sci Rep. 2023 Nov 30;13:21114. doi: 10.1038/s41598-023-47805-2 (PMC10689793; doi:10.1038/s41598-023-47805-2)
Supplement: Supplementary file 1 — Supplementary Figure 1. [file 41598_2023_47805_MOESM1_ESM.pdf]

A deep learning model predicts cancer types using circulating tumor cells.

Somayah Albaradei*[1], Nofe Alganmi*[1,2], Abdulrahman Albaradie[3], Eaman Alharbi[1], Olaa Motwalli[4], Maha A. Thafar[5], Magbubah Essack[#6,7], Xin Gao[#6,7]

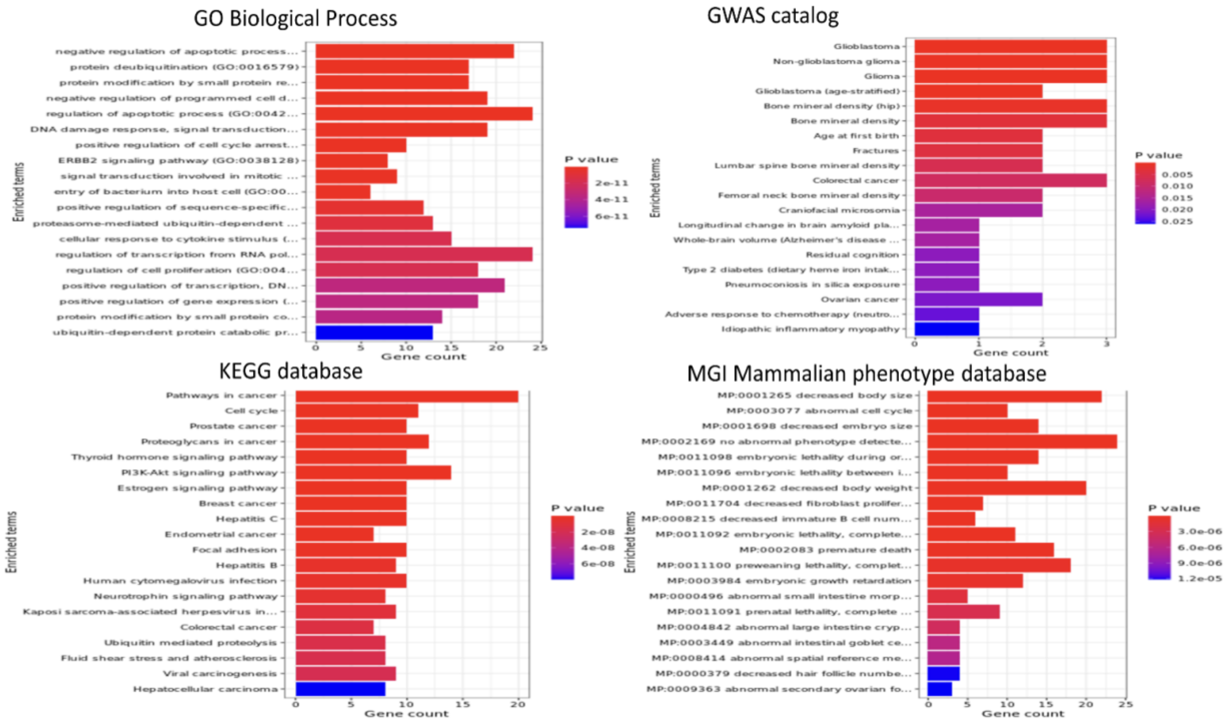

Figure.1 The bar plots for enrichment analysis including the top 20 significant GO terms detected from each database.
